# Characterization of self-assembled virus-like particles of rat hepatitis E virus generated by recombinant baculoviruses

**DOI:** 10.1099/vir.0.034835-0

**Published:** 2011-12

**Authors:** Tian-Cheng Li, Kumiko Yoshimatsu, Shumpei P. Yasuda, Jiro Arikawa, Takaaki Koma, Michiyo Kataoka, Yasushi Ami, Yuriko Suzaki, Le Thi Quynh Mai, Nguyen Thuy Hoa, Tetsu Yamashiro, Futoshi Hasebe, Naokazu Takeda, Takaji Wakita

**Affiliations:** 1 Department of Virology II, National Institute of Infectious Diseases, Gakuen 4-7-1, Musashi-murayama, Tokyo 208-0011, Japan; 2 Department of Microbiology, Graduate School of Medicine, Hokkaido University, Kita-15, Nishi-7, Kita-ku, Sapporo 060-8638, Japan; 3 Department of Pathology, National Institute of Infectious Diseases, Gakuen 4-7-1, Musashi-murayama, Tokyo 208-0011, Japan; 4 Division of Experimental Animals Research, National Institute of Infectious Diseases, Gakuen 4-7-1, Musashi-murayama, Tokyo 208-0011, Japan; 5 National Institute of Hygiene and Epidemiology, Hanoi, Vietnam, No. 1 Yersin Street, Hai Ba Trung District, Hanoi, Vietnam; 6 Center for Infectious Disease Research in Asia and Africa, Nagasaki University, 1-12-4 Sakamoto, Nagasaki 852-8523, Japan; 7 Center of International Collaborative Research, Nagasaki University, 1-12-4 Sakamoto, Nagasaki 852-8523, Japan; 8 Research Collaboration Center on Emerging and Re-Emerging Infections, Building 10, National Institute of Health, Department of Medical Sciences, Ministry of Public Health, Tivanond 14 Road, Muang, Nonthaburi 11000, Thailand

## Abstract

Hepatitis E virus (HEV) is a causative agent of hepatitis E. Recently, a novel hepatitis E-like virus was isolated from Norway rats in Germany. However, the antigenicity, pathogenicity and epidemiology of this virus are unclear because of the lack of a cell-culture system in which to grow it. In this study, an N-terminally truncated ORF2 protein was expressed in insect Tn5 cells using a recombinant baculovirus expression system and a large amount of 53 kDa protein was expressed and efficiently released into the supernatant. Electron microscopic analyses of the purified 53 kDa protein revealed that the protein self-assembled into two types of empty HEV-like particles (rat HEVLPs). The smaller rat HEVLPs were estimated to be 24 nm in diameter, which is similar to the size of genotype G1, G3 and G4 HEVLPs. The larger rat HEVLPs were estimated to measure 35 nm in diameter, which is similar to the size of native rat HEV particles. An ELISA to detect antibodies was established using rat HEVLPs as the antigens, which demonstrated that rat HEVLPs were cross-reactive with G1, G3 and G4 HEVs. Detection of IgG and IgM antibodies was performed by examination of 139 serum samples from wild rats trapped in Vietnam, and it was found that 20.9 % (29/139) and 3.6 % (5/139) of the samples were positive for IgG and IgM, respectively. In addition, rat HEV RNA was detected in one rat serum sample that was positive for IgM. These results indicated that rat HEV is widespread and is transmitted among wild rats.

## Introduction

Hepatitis E virus (HEV) is the causative agent of hepatitis E, a viral disease that manifests as acute hepatitis (Emerson & Purcell, 2003). The disease represents an important public health problem in developing countries and is transmitted primarily by the faecal–oral route (Balayan *et al.*, 1983). In developed countries, a number of sporadic cases have been described, and the disease is primarily transmitted in a zoonotic fashion (Meng, 2010). HEV is a positive-sense ssRNA virus that belongs to the genus *Hepevirus* in the family *Hepeviridae* (Emerson *et al.*, 2005). The HEV genome is approximately 7.2 kb, containing a 5′ non-coding region (27–35 nt) followed by three overlapping ORFs and a 3′ non-coding region of approximately 65–74 nt followed by a poly(A) tail. ORF1 at the 5′ end of the genome encodes several non-structural proteins, whilst ORF2 encodes an immunodominant capsid protein (Jameel, 1999). ORF3, which partially overlaps with ORF2, encodes a cytoskeleton-associated phosphoprotein with multiple functions (Korkaya *et al.*, 2001; Meng *et al.*, 1997; Zafrullah *et al.*, 1997).

To date, at least four genotypes of HEV, G1–G4, have been isolated from humans, and G3 and G4 HEVs have been isolated from pigs, wild boar and wild deer (Bradley & Balayan, 1988; Huang *et al.*, 1992; Meng *et al.*, 1997; Takahashi *et al.*, 2001, 2004; Wang *et al.*, 2000). Recent evidence has indicated that G3 and G4 HEVs are transmitted from wild boar and wild deer to humans by zoonosis (Li *et al.*, 2005a; Tei *et al.*, 2003). Even more recently, new HEV strains (G5 and G6 HEVs) have been identified in wild boar, and other HEV-like viruses have been identified in rabbits and rats (Johne *et al.*, 2010a; Zhao *et al.*, 2009). Rat HEV shares little sequence identity with G1–G4 HEVs discovered thus far, suggesting that there are additional HEV-like viruses in other animal species (Johne *et al.*, 2010b).

To date, the entire rat genome sequence has been determined using two rat HEVs and it has been demonstrated that the genome contains three major ORFs, ORF1–3, similar to the genomes of G1–G4 HEVs (Johne *et al.*, 2010a). However, the antigenicity, pathogenicity and epidemiology of this virus remain unclear because of the lack of a viable cell-culture system in which to grow the virus.

In this study, we describe the efficient expression of N-terminally truncated rat HEV ORF2 protein with a synthetic gene derived from a German rat HEV strain isolated in 2010 (Johne *et al.*, 2010a). The viral protein, expressed by a recombinant baculovirus in insect Tn5 cells, was found to self-assemble into virus-like particles (VLPs), which were then efficiently released into the culture medium. The VLPs exhibited antigenic cross-reactivity with G1, G3 and G4 HEVs. An ELISA was developed using rat HEV-like particles (HEVLPs) as antigen and used to examine rat HEV-specific IgG and IgM responses. The antibody prevalence indicated that rat HEV is widespread among wild rats in Vietnam.

## Results

### Expression of rat HEV ORF2 in insect cells

BTL-Tn-5B1-4 (Tn5) cells were infected at an m.o.i. of 10 with recombinant baculoviruses Ac[ORF2] and Ac[ΔORF2] containing the full-length and N-terminal 100 aa-deleted ORF2 of rat HEV, and the infected cells were incubated at 26.5 °C to express the full-length ORF2 and N-terminally truncated ORF2. The cells were harvested daily up to day 10 post-infection (p.i.), and the proteins generated in the infected cells and supernatant were analysed by Western blotting. In Ac[ORF2]-infected Tn5 cells, a protein band with a molecular mass of 69 kDa (p69) appeared at 2 days p.i., and reached a peak on day 3 p.i. The molecular mass of p69 was in agreement with that calculated for the full-length rat ORF2; however, p69 was not detected in the supernatant (data not shown).

**Fig. 1.**
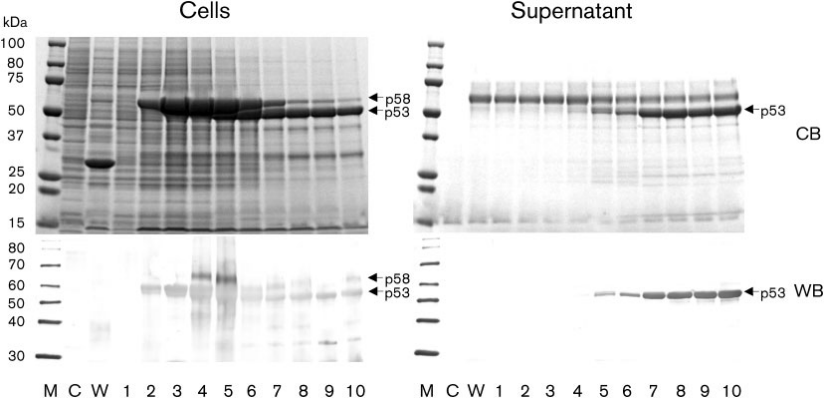
Time course of the expression of N-terminal 100 aa-truncated rat HEV ORF2. Tn5 cells were infected with recombinant baculovirus Ac[ΔORF2], incubated at 26.5 °C and harvested on days 1–10 p.i. (lanes 1–10). Five microlitres of the culture medium or lysate from 10^5^ cells was analysed by SDS-PAGE. Protein bands were visualized by Coomassie blue staining (CB) and Western blotting with anti-G1 HEVLP rabbit serum (WB). M, Molecular mass marker; C, mock-infected control; W, wild-type baculovirus-infected cells.

In the Ac[ΔORF2]-infected Tn5 cells, a major protein with a molecular mass of 58 kDa (p58) was detected in the cells on day 2 p.i., and expression levels reached a peak at day 4 p.i. (Fig. 1). A protein migrating with a molecular mass of 53 kDa (p53) was found in the cells on day 5 p.i., and reached a peak on days 7–10 p.i. in the supernatant. These p58 and p53 proteins were synthesized only in Ac[ΔORF2]-infected cells, and not in the mock-infected or wild-type baculovirus-infected cells. The p58 and p53 proteins reacted with anti-G1 HEVLP antibody in Western blots (Fig. 1).

### Self-assembly of the recombinant N-terminal 100 aa-deleted ORF2 protein

**Fig. 2.**
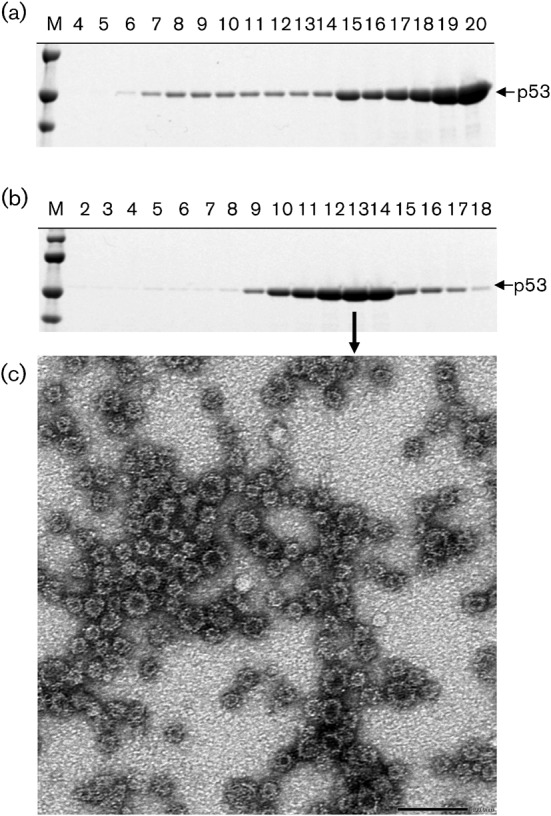
Purification of rat HEVLPs. (a, b) The supernatant of recombinant baculovirus-infected Tn5 cells was centrifuged for 3 h at 32 000 r.p.m. in a Beckman SW32Ti rotor. The pellet was resuspended in 4.5 ml or 100 µl EX-CELL 405 and purified by CsCl-gradient (a) or sucrose-gradient (b) centrifugation, respectively. Aliquots from the gradient were analysed by SDS-PAGE (5–20 % acrylamide gradient) and stained with Coomassie blue. (c) To examine the HEVLPs, each fraction containing p58 protein was stained with 2 % uranyl acetate and observed by electron microscopy. Bar, 100 nm.

The culture medium of Ac[ΔORF2]-infected Tn5 cells was harvested at 7 days p.i., and p53 was purified by CsCl-gradient centrifugation as described in Methods. The p53 protein was broadly distributed in fractions 5–20, but mainly in fractions 19 and 20 (Fig. 2a). However, no HEVLPs were observed by electron microscopy (data not shown). When separated by a sucrose gradient, the p53 protein was distributed primarily in fractions 12–14, all of which showed a mean density of 1.100 g ml^−3^ (Fig. 2b). To identify the p53 protein, the N-terminal amino acid sequence was determined by microsequencing and the sequence AQAPAPNTAP was obtained. This sequence is identical to aa 101–110 of rat HEV ORF2, indicating that p53 was derived from the rat HEV ORF2 protein. Because the molecular mass of the 100 aa-deleted rat HEV ORF2 protein was 58 kDa, the p53 protein is processed from p58, presumably by a deletion at the C terminus. Observation of fraction 13 by electron microscopy revealed two sizes of spherical particles with respective diameters of 24 and 35 nm (Fig. 2c). The morphology of these small particles was similar to that of G1, G3 and G4 HEVLPs produced by recombinant baculoviruses harbouring N-terminal 111 aa-deleted HEV ORF2 (Guu *et al.*, 2009; Li *et al.*, 1997; Yamashita *et al.*, 2009). The size of the 35 nm particles was the same as that of the native rat HEV particles. The yield of the purified rat HEVLPs reached 1.5 mg per 10^7^ Tn5 cells. To determine whether nucleic acids were packaged into rat HEVLPs, nucleic acids were extracted from purified rat HEVLPs and analysed by agarose gel electrophoresis. However, we could not detect any nucleic acids in rat HEVLPs (data not shown). These results indicated that the p53 protein self-assembled into VLPs and demonstrated that the ORF2 gene encoded the rat HEV capsid protein. No HEVLPs were obtained from either Ac[ΔORF2]-infected or Ac[ORF2]-infected *Spodoptera frugiperda*-derived (Sf9) cells (data not shown).

### Antigenic cross-reactivity among rat, G1, G3 and G4 HEVs

**Fig. 3.**
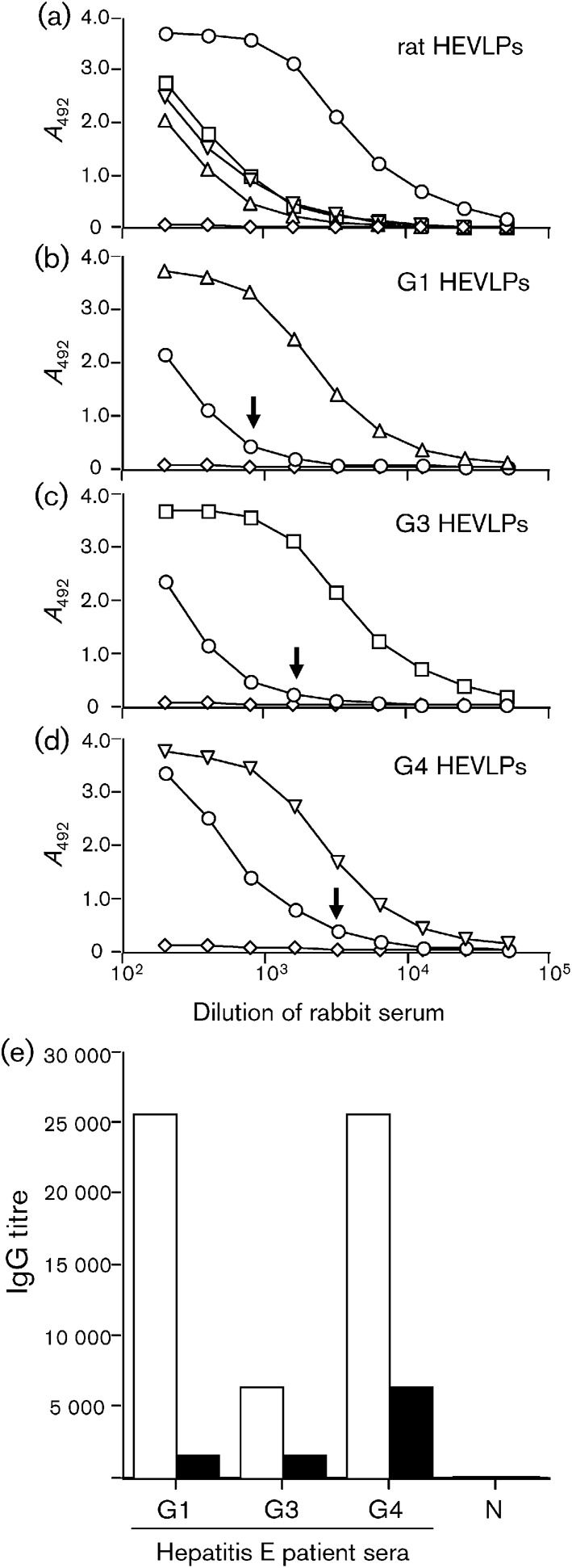
Antigenic cross-reactivity among rat, G1, G3 and G4 HEVLPs. (a–d) The *A*_492_ values of hyperimmune sera from rabbits immunized with rat (○; a), G1 (▵; b), G3 (□; c) or G4 (▽; d) HEV and of pre-immunized rabbit serum (◊) were determined by antibody ELISA using the four VLP antigens indicated. Arrows indicate the end-point titres against anti-rat HEVLP serum. (e) Antigenicity of rat HEVLPs. The IgG titres in serum samples from G1, G3 and G4 hepatitis E patients or serum from a healthy individual (N) were determined by antibody ELISA using rat HEVLPs (filled bars) or G1 HEVLPs (open bars) as the antigen.

The rat HEV capsid protein p53 reacted with anti-G1 HEV antibody, as determined by Western blotting (Fig. 2), which suggested that the rat HEV had a similar antigenicity to G1 HEV. The antigenic cross-reactivity among rat, G1, G3 and G4 HEVs was examined by ELISA. For this purpose, rabbits were immunized with rat, G1, G3 or G4 HEVLPs. Three weeks after injection, all of the rabbits elicited high levels of IgG antibodies against the homologous antigen (HEVLPs), with ELISA titres reaching 1 : 25 600 (rat), 1 : 12 800 (G1), 1 : 12 800 (G3) and 1 : 25 600 (G4) (Fig. 3a–d). The anti-rat HEVLP antibody reacted with not only homologous rat HEVLPs (Fig. 3a) but also with heterologous G1, G3 and G4 HEVLPs with titres of 1 : 800, 1 : 1600 and 1 : 3200, respectively (Fig. 3b–d, arrows). Conversely, the antibodies against G1, G3 and G4 HEVLPs were cross-reactive with rat HEVLPs (Fig. 3a). The antigenic cross-reactivity was confirmed by an antibody ELISA using rat HEVLPs and serum from convalescent hepatitis E patients. As depicted in Fig. 3(e), rat HEVLPs showed cross-reactivity with sera from G1, G3 and G4 hepatitis E patients, although the titres were lower than those detected using G1 HEVLPs as antigen. These results indicated that rat HEV has antigenic epitope(s) in common with those of G1, G3 and G4 HEVs.

### Prevalence of IgG and IgM antibodies in wild rats

**Fig. 4.**
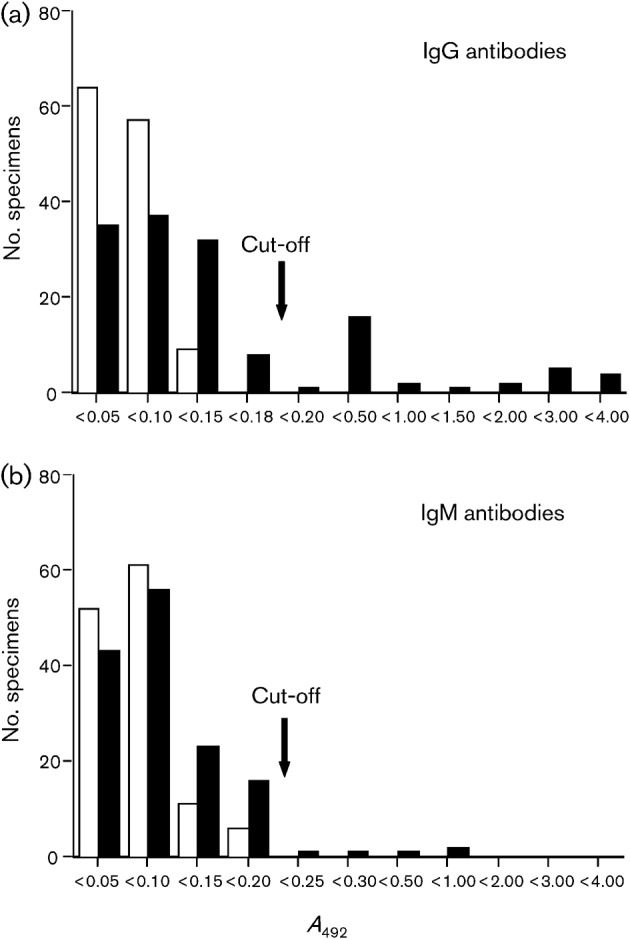
Detection of anti-rat HEV IgG and IgM antibodies in laboratory and wild rats. Serum samples were collected from laboratory rats in Japan and from wild rats in Vietnam. Anti-HEV IgG and IgM antibodies were detected by antibody ELISA with 1 : 200-diluted sera. Open bars, laboratory rats; filled bars, wild rats.

In order to detect IgG and IgM antibodies against rat HEV, ELISAs were developed as follows. A total of 130 serum samples from laboratory rats were used at a dilution of 1 : 200 to determine the cut-off value for the ELISA. The absorbance values at 492 nm (*A*_492_) of the IgG of these serum samples were between 0.016 and 0.147, with a mean value±sd of 0.052±0.043. The cut-off value for IgG was set at 0.181, 3 sd above the mean *A*_492_ value (Fig. 4a). Similarly, the *A*_492_ values of the IgM of these serum samples were between 0.021 and 0.178, with a mean of 0.061±0.050. The cut-off value for IgM was set at 0.211, 3 sd above the mean *A*_492_ value (Fig. 4b).

A total of 139 serum samples collected from wild rats in Vietnam were examined, and 20.9 % (29/139) of the samples were found to be positive for IgG antibody, whereas 3.6 % (5/139) were positive for IgM antibody (Fig. 4). All five of the IgM-positive serum samples were also positive for IgG. Among the animals trapped in Vietnam, 75 were from Haiphong and 64 were from Hanoi. The rate of positivity for anti-rat HEV IgG was 22.7 % (17/75) for the samples from Haiphong, and 18.8 % (12/64) for the samples from Hanoi. The rate of positivity did not differ significantly between samples from these two areas (*P*>0.05). Among the 139 rat serum samples, 16 were from *Rattus tanezumi* and 123 from *Rattus norvegicus*; the IgG-positive rates were 25.0 % (4/16) and 20.3 % (25/123), respectively. The IgG-positive rates were not significantly different between *R. tanezumi* and *R. norvegicus* (*P*>0.05). These results suggested that rat HEV infection is widespread and that transmission is ongoing among wild rats in Vietnam.

### Detection of the rat HEV genome by RT-PCR

**Fig. 5.**
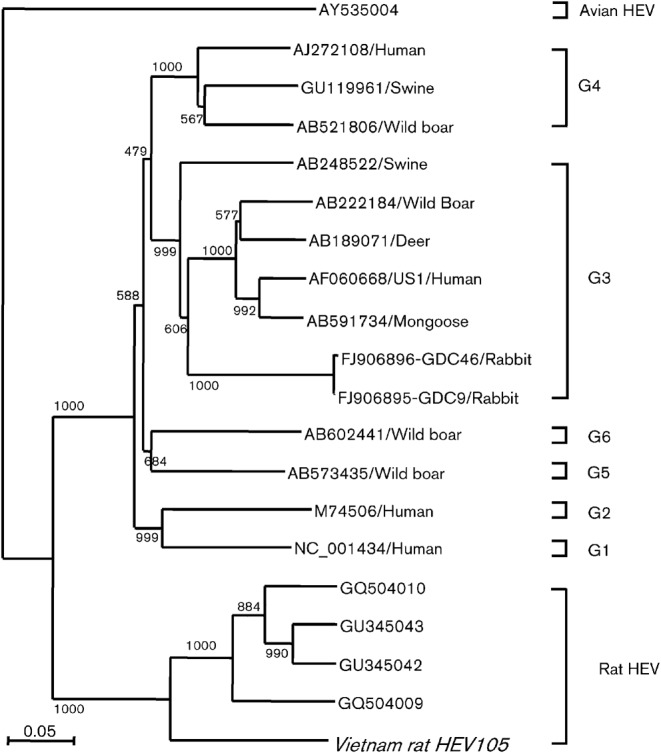
Phylogenetic analysis of rat HEV based on the partial nucleotide sequence of the Vietnam rat HEV 105 strain (901 nt) using avian HEV as an outgroup. Bootstrap values were determined based on 1000 resamplings of the datasets and are shown at the nodes.

The IgM-positive serum samples were selected to detect rat HEV RNA using a nested broad-spectrum RT-PCR, and one serum sample was found to be positive for rat HEV. A total of 901 nt corresponding to nt 4108–5008 of the rat HEV genome (GenBank accession no. GU345042) comprising the C terminal ORF1 (814 nt), the junction region (27 nt) and the N terminal ORF2 (60 nt) were compared with the corresponding sequences of other HEVs. A phylogenetic analysis based on these 901 nt indicated that the Vietnamese rat strain formed a cluster with other rat HEVs (Fig. 5). This strain was designated Vietnam rat HEV 105. The nucleotide identity between Vietnam rat HEV 105 and the four German rat HEV strains was 78.18–79.43 %. The identity of the deduced 270 aa of the ORF1 C terminus was 94.07–92.96 %, demonstrating considerable differences among strains. Because the nucleotide identity between the Vietnamese and German strains was <80 %, the Vietnam rat HEV 105 strain may belong to a new genotype of rat HEV.

## Discussion

Rat HEV is a new genotype of HEV, and nucleotide sequence identities with HEV G1–4 were 55.1–55.9 %. Because no cell-culture system has yet been developed for rat HEV, it remains necessary to express the capsid protein and generate VLPs in order to analyse the antigenicity and immunogenicity of this pathogen; these recombinant molecules are also extremely useful for seroepidemiological studies of rat HEV infection in wild rats.

For the production of VLPs, the full-length rat HEV ORF2 was initially expressed by a recombinant baculovirus; however, the recombinant protein derived from this gene was not released into the culture supernatant and did not form VLPs. In the case of G1, G3 and G4 HEVs, an N terminal 111 aa-deleted ORF2 protein has been found to be released efficiently into the supernatant and to self-assemble into VLPs. Therefore, we employed the same strategy for the current analysis. When the deduced amino acid sequence of the rat HEV ORF2 (GenBank accession no. GU345042) was aligned with that of representatives of HEV G1, G3 and G4 (GenBank accession nos DQ079624, DQ079627 and DQ079631, respectively), we found that aa 101 in the rat HEV ORF2 corresponded to aa 112 in the G1, G3 and G4 HEV ORF2. Therefore, we expressed the N-terminal 100 aa-deleted rat HEV ORF2 using a recombinant baculovirus expression system. As expected, the recombinant protein, p53, was released into the supernatant and formed VLPs (Fig. 1). Deletion of 100 aa from the N terminus of ORF2 was essential for the formation of rat HEV VLPs. Although we attempted to express the full-length and N-terminal 100 aa-deleted rat HEV ORF2 in Sf9 cells, another insect cell line, the recombinant protein was not released into the supernatant and no VLPs were detected. These characteristics are identical to those observed with G1, G3 and G4 HEVs, indicating that processing of the recombinant protein and the formation of VLPs are both cell-dependent events.

In previous studies of G1, G3 and G4 HEVLPs, the VLPs were purified by CsCl-gradient centrifugation and were concentrated in the fraction with a density of 1.285 g cm^−3^ (Guu *et al.*, 2009; Li *et al.*, 1997; Yamashita *et al.*, 2009). In contrast, in the present study, the rat HEV p53 was broadly separated and no particles were visible after CsCl-gradient centrifugation. However, a large amount of purified rat HEVLPs was obtained by sucrose-gradient centrifugation, indicating that rat HEVLPs are unstable at high concentrations of CsCl. In previous studies, only small empty particles with a diameter of 24 nm were detected in the cells and supernatant when the N-terminal 111 aa-deleted G1, G3 and G4 HEV ORF2s were expressed in the recombinant baculovirus (Guu *et al.*, 2009; Li *et al.*, 1997; Yamashita *et al.*, 2009). When the N-terminal 100 aa-deleted rat HEV ORF2 was expressed, two VLPs with respective diameters of 24 and 35 nm were visible. At the present time, there is no explanation for this finding and therefore further studies including three-dimensional structural analysis are needed.

When rabbits were immunized with rat HEVLPs, a strong immune response was induced, with high IgG titres and in the absence of any adjuvant or booster injections, suggesting that rat HEVLPs are highly immunogenic in rabbits. Rat HEVLPs were cross-reactive with antibodies elicited in acute hepatitis E patients; moreover, the antibodies induced by rat HEVLPs were cross-reactive with G1, G3 and G4 HEVLPs. These results clearly demonstrated that rat HEV and G1, G3 and G4 HEVs share at least one common epitope. HEVLPs are composed of a single capsid protein, which folds into three major domains: the shell (S) domain, the middle (M) domain and the protruding (P) domain. The outer surface of the particles, which is a target for antibodies, is formed primarily by the M and P domains (Xing *et al.*, 2010; Yamashita *et al.*, 2009). The amino acid identities of the full-length capsid protein (1–660 aa), S domain (118–308 aa), M domain (309–444 aa) and partial P domain (528–556 aa) between rat HEV (GenBank accession no. GU354042) and the G1, G3 and G4 HEVs were found to be 50.5–51.2, 75.4–76.4, 66.2–67.6 and 75.9–79.3 %, respectively. The amino acid identities of the S, M and partial P domains of each group were clearly higher than those of the other capsid regions, which suggests that common epitope(s) may be present in the M and/or P domains on the surface of the particles.

A high prevalence of anti-HEV antibody has been reported in wild rats in the USA and Japan (Favorov *et al.*, 2000; Hirano *et al.*, 2003; Kabrane-Lazizi *et al.*, 1999). In samples from these countries, antibodies to rat HEV were detected by ELISA using antigens derived from G1 HEV isolated in Pakistan or Myanmar. As this is only indirect evidence of the cross-reactivity between rat and G1 HEVs, it will be necessary to examine the prevalence of anti-rat HEV antibodies in wild rats using homologous antigens, i.e. rat HEVLPs, which may exhibit stronger reactivity than the heterologous antigens, i.e. G1 HEVLPs. The ELISA method developed in this study will be useful for monitoring the circulation of rat HEV in wild rats.

## Methods

### 

#### Construction of a transfer vector.

The full-length ORF2 of rat HEV containing a *Bam*HI site before the start codon and an *Xba*I site after the stop codon was synthesized based on the rat HEV sequence deposited in GenBank (GenBank accession no. GU345042). The full-length ORF2 was then was cloned into the vector pUC57 (GeneScript) to generate the plasmid pUC57-rat-ORF2. A DNA fragment encoding the N-terminal 100 aa-truncated rat HEV ORF2 was amplified by PCR using pUC57-rat-ORF2 with forward primer rat-E-F2 (5′-AAGGATCCATGGCACAGGCACCGGCGCCTA-3′) and reverse primer rat-E-R1 (5′-ATCTAGATCAGACACTATCGGCGGCTGCTG-3′). The amplified DNA fragment was purified using a PCR purification kit (Qiagen). The full-length and N-terminal 100 aa-truncated ORF2 were digested with *Bam*HI and *Xba*I and ligated into a baculovirus transfer vector, pVL1393 (Pharmingen), to yield plasmids pVL1393-ORF2 and pVL1393-ΔORF2, respectively.

#### Construction of a recombinant baculovirus and expression of capsid proteins.

Sf9 cells (RIKEN Cell Bank) were co-transfected with linearized wild-type *Autographa californica* nuclear polyhedrosis virus DNA (BaculoGold 21100D; Pharmingen) and either pVL1393-ORF2 or pVL1393-ΔORF2 by a Lipofectin-mediated method as specified by the manufacturer (Gibco-BRL). The cells were incubated at 26.5 °C in TC-100 medium (Gibco-BRL) supplemented with 8 % FBS and 0.26 % bactotryptose phosphate broth (Difco Laboratories). The recombinant virus was plaque purified three times in Sf9 cells and designated Ac[ORF2] and Ac[ΔORF2], respectively. To achieve large-scale expression, an insect cell line from *Trichoplusia ni*, Tn5 (Invitrogen), was used (Wickham & Nemerow, 1993). Tn5 cells were infected with Ac[ORF2] or Ac[ΔORF2] at an m.o.i. of 10, and the cells were cultured in EX-CELL 405 medium (JRH Biosciences) at 26.5 °C, as described previously (Li *et al.*, 2005b, 1997). VLPs of G1, G3 and G4 HEVs were produced as described previously (Guu *et al.*, 2009; Li *et al.*, 1997; Yamashita *et al.*, 2009).

#### SDS-PAGE and Western blot analysis.

The proteins in the cell lysates and culture medium were separated by SDS-PAGE with a 5–20 % acrylamide gradient gel and stained with Coomassie blue. For Western blot analysis, proteins in the gel were electrophoretically transferred onto a nitrocellulose membrane. The membrane was then soaked with 5 % skimmed milk in 50 mM Tris/HCl (pH 7.4), 150 mM NaCl, and incubated with a rabbit anti-G1 HEVLP polyclonal antibody as described previously (Li *et al.*, 1997). Detection of the rabbit IgG antibody was achieved using alkaline phosphatase-conjugated goat anti-rabbit antibody (1 : 1000 dilution; Chemicon International). Nitro blue tetrazolium chloride and 5-bromo-4-chloro-3-indolyl phosphate *p*-toluidine were used for detection of antibody binding (Bio-Rad Laboratories).

#### Purification of rat HEVLPs.

Recombinant baculovirus-infected Tn5 cells were harvested on day 7 p.i. The intact cells, cell debris and progeny baculoviruses were removed by centrifugation at 10 000 ***g*** for 60 min. The supernatant was then spun at 32 000 r.p.m. for 3 h in a Beckman SW32Ti rotor, and the resulting pellet was resuspended in EX-CELL 405 medium at 4 °C overnight. For sucrose-gradient centrifugation, 1 ml of each sample was laid on top of a 10–40 % (w/w) gradient and centrifuged at 32 000 r.p.m. for 2 h in a Beckman SW55Ti rotor. For CsCl-gradient centrifugation, 4.5 ml of each sample was mixed with 2.1 g CsCl and centrifuged at 35 000 r.p.m. for 24 h at 10 °C in the same rotor. The gradient was fractionated into 250 µl aliquots, and each fraction was weighed to estimate the buoyant density and isopycnic point. Each fraction was diluted with EX-CELL 405 medium and centrifuged for 2 h at 50 000 r.p.m. in a Beckman TLA55 rotor to sediment the HEVLPs.

#### Electron microscopy.

Purified HEVLPs were placed on a carbon-coated grid for 45 s, rinsed with distilled water, stained with a 2 % uranyl acetate solution and examined under a JEOL TEM-1400 electron microscope operating at 80 kV.

#### N-terminal amino acid sequence analysis.

The proteins separated by SDS-PAGE were visualized by staining with GelCode Blue Staining Reagent (Pierce) and purified by sucrose-gradient centrifugation. N-terminal amino acid microsequencing was carried out using 100 pmol protein by Edman automated degradation on an Applied Biosystems Model 477 Protein Sequencer.

#### Hyperimmune sera.

Rabbits were immunized with rat, G1, G3 and G4 HEVLPs. Immunization was performed by one percutaneous injection of purified HEVLPs with a dose of 500 µg per rabbit. Rats were immunized with the recombinant rat HEVLPs by intramuscular injection at a dose of 200 µg per rat, and booster injections were carried out at 4 and 6 weeks after the first injection with half doses of rat HEVLPs. All of the injections, including booster injections, were carried out without adjuvant. Immunized animals were bled 3 weeks after the last injection.

#### Rat serum samples.

A total of 130 serum samples from laboratory rats (Wistar; Japan SLC) were collected at the Division for Experimental Animal Research of the National Institute of Infectious Diseases of Japan. A total of 139 serum samples from wild rats were collected in Vietnam (39 samples were collected in 2009 in Haiphong, and 64 and 36 sera were collected in Hanoi and Haiphong in 2011, respectively). With regard to the rat species sampled, 123 were identified as *R. norvegicus* and 16 were identified as *R. tanezumi*. All of the serum samples were stored at −80 °C until use.

#### Detection of IgG and IgM antibodies.

Flat-bottomed 96-well polystyrene microplates (Immulon 2; Dynex Technologies) were coated with the purified rat HEVLPs (1 µg ml^−1^, 100 µl per well) and incubated overnight at 4 °C. Unbound HEVLPs were removed and the plates washed twice with 10 mM PBS containing 0.05 % Tween 20 (PBS-T) and then blocked with 200 µl 5 % skimmed milk (Difco Laboratories) dissolved in PBS-T for 1 h at 37 °C. After the plates had been washed four times with PBS-T, diluted rat serum samples (100 µl per well) were added in duplicate. The plates were incubated at 37 °C for 1 h and then washed three times as described above. The wells were incubated with 100 µl HRP-conjugated goat anti-rat IgG (H+L) (1 : 10 000 dilution; Zymed Laboratories) or HRP-conjugated goat anti-rat IgM (1 : 100 000 dilution; Jackson ImmunoResearch Laboratories) diluted in PBS-T containing 1 % skimmed milk. The plates were incubated at 37 °C for 1 h and washed four times with PBS-T. One hundred microlitres of substrate orthophenylenediamine (0.4 mg ml^−1^; Sigma Chemical) and 5 µl H_2_O_2_ (30 % in 12.5 ml substrate buffer) were added to each well. The plates were incubated in a dark room at room temperature for 30 min, and then 50 µl 2 M H_2_SO_4_ was added to each well. Absorbance was measured at 492 nm. The cut-off values for IgG and IgM were determined as described previously (Li *et al.*, 2000). A sample was considered to be positive when the absorbance exceeded the cut-off value. Pre-immunization and rat HEVLP-immunized rat sera were used as the negative and positive controls, respectively. Detection of human and rabbit anti-HEV IgG was performed as described previously (Li *et al.*, 1997).

#### RNA extraction and nested broad-spectrum RT-PCR.

Total RNA was extracted using a QIAamp viral RNA mini kit (Qiagen) and resuspended in 20 µl DNase-, RNase- and proteinase-free water. Reverse transcription was performed at 42 °C for 50 min, followed by 70 °C for 15 min in a 20 µl reaction mixture containing 1 µl Superscript II RNase H^−^ reverse transcriptase (Invitrogen), 1 µl oligo(dT) primer, 1 µl RNaseOUT (Invitrogen), 2 µl 0.1 M DTT, 4 µl 5× RT buffer (Invitrogen), 1 µl 10 mM dNTPs, 5 µl RNA and 5 µ1 distilled water.

A nested broad-spectrum RT-PCR analysis was performed to amplify a portion of ORF1, based on a method described previously with slight modifications (Johne *et al.*, 2010b). Five microlitres of the cDNA was used for the first PCR in a 50 µl reaction volume containing an external forward primer, HEV-cs (5′-TCGCGCATCACMTTYTTCCARAA-3′), and an external reverse primer, HEV-cas (5′-GCCATGTTCCAGACDGTRTTCCA-3′). Each cycle consisted of denaturation at 95 °C for 30 s, primer annealing at 52 °C for 45 s and extension at 72 °C for 60 s, followed by final extension at 72 °C for 7 min. Two microlitres of the first PCR product was used for the nested PCR with an internal forward primer, HEV-csn (5′-TGTGCTCTGTTTGGCCCNTGGTTYCDG-3′), and an internal reverse primer, HEV-casn (5′-CCAGGCTCACCRGARTGYTTCTTCCA-3′). Each cycle consisted of 95 °C for 30 s, 55 °C for 45 s and 72 °C for 60 s, followed by 72 °C for 7 min. The nested PCR products were separated by electrophoresis on 2 % agarose gels.

To amplify the Vietnam rat HEV genome, two pairs of primers were designed. The forward primers were designed according to the Vietnamese rat HEV strain (GenBank accession no. JN040433). The reverse primers were designed according to the German rat HEV strain (GenBank accession no. GU354042). The first PCR analysis was carried out in a 50 µl volume reaction mixture with an external forward primer, rat-HEV-F10 (5′-GAAGGCCATAGTCGCCAACCTG-3′, nt 4117–4138), and an external reverse primer, rat-HEV-R7 (5′-TCAGACACTATCGGCGGCTG-3′, nt 6864–6883). Each cycle consisted of 95 °C for 30 s, 55 °C for 60 s and 72 °C for 4 min, followed by 72 °C for 7 min. Two microlitres of the first PCR product was used for the nested PCR with an internal forward primer, HEV-F11 (5′-AAGGCGTGCAGAGTGTTTGAGA-3′, nt 4205–4226), and an internal reverse primer, rat-HEV-R9 (5′-CGGGCTCCACCGGGGTACAT-3′, nt 5013–5032). Each cycle consisted of 95 °C for 30 s, 55 °C for 45 s and 72 °C for 60 s, followed by 72 °C for 7 min.

Nucleotide sequencing of the PCR products was carried out using primers HEV-csn, HEV-casn, HEV-F11 and HEV-R9 on an ABI 3130 Genetic Analyzer automated sequencer (Applied Biosystems) and a BigDye Terminator Cycle Sequencing Ready Reaction kit (Applied Biosystems) according to the manufacturer’s instructions.

#### Statistical analysis.

Comparisons of the rate of positivity between different areas and between *R. norvegicus* and *R. tanezumi* were performed with an unpaired Student’s *t*-test.
